# Morphological and transcriptional insights into the role of histone phosphorylation-related genes in early development of the chicken duodenum

**DOI:** 10.5713/ab.25.0108

**Published:** 2025-06-10

**Authors:** Xiaofeng Li, Bing Yang

**Affiliations:** 1 College of Animal Science and Technology, Ningxia University, Yinchuan, China; 2 Key Laboratory of Embryo Development and Reproductive Regulation, Fuyang, China; 3 College of Animal Science, Anhui Science and Technology University, Bengbu, China; 4 National Key Laboratory for Tea Plant Germplasm Innovation and Resource Utilization, Anhui Agricultural University, Hefei, China; 5 Longyan University & Fujian Provincial Key Laboratory for Prevention and Control of Animal Infectious Diseases and Biotechnology, Longyan University, Longyan, China

**Keywords:** Chicken, Duodenum Development, Histone Phosphorylation, Hub Genes

## Abstract

**Objective:**

This study aimed to investigate the morphological transitions and the role of histone phosphorylation-related genes during the first week of duodenal development in broilers.

**Methods:**

At hatch (D0) and 7 days post-hatch (D7), five broiler chickens were humanely euthanized and duodenal samples were collected to assess the histomorphology, specifically the duodenal villus height (VH), crypt depth (CD), and the VH/CD ratio. The gene expression data of duodenum of broilers at D0 and D7 obtained from the GEO database. Differentially expressed genes (DEG) analyses were conducted using GEO2R. In addition, histone phosphorylation-related genes were obtained from the GeneCards database. Function enrichment for DEGs was conducted using the DAVID and PANTHER databases. Hub genes were identified using the CytoHubba plugin in Cytoscape, employing four different methods: MCON, DNMC, EPC, and MNC. Statistical analyses were conducted using IBM SPSS Statistics.

**Results:**

We identified striking developmental transformations: VH surged by 63.02% (p<0.05) and VH/CD ratio doubled (100.68% increase, p<0.05), accompanied by 17.81% CD reduction (p<0.05). Transcriptomic profiling revealed 449 histone phosphorylation-related DEGs, comprising 163 up-regulated and 286 down-regulated candidates. Functional enrichment analysis suggested that these genes participated in phosphorylation, intracellular protein phosphorylation, protein dephosphorylation, immune response, as well as MAPK, PPAR, ErbB, and adipocytokine pathways. Importantly, we identified eight hub genes orchestrating duodenal maturation, including *LGALS3*, *ITGB2*, *IRF7*, *SOCS3*, and *CSF1R*, *KIF23*, *SMC2*, and *DLGAP5*.

**Conclusion:**

These findings establish a novel paradigm wherein histone phosphorylation coordinates intestinal morphogenesis, providing mechanistic insights for optimizing poultry intestinal health and nutritional strategies.

## INTRODUCTION

Early duodenum development plays a vital role in the overall health and performance of chickens [1]. As the primary site for nutrient absorption, the duodenum significantly influences feed efficiency, immune response, and production performance [2]. A well-developed duodenum is crucial for optimizing the utilization of feed, which directly impacts growth rates and feed conversion ratios [3]. Proper nutrient absorption in the duodenum ensures that essential proteins, carbohydrates, and fats are adequately assimilated, leading to improved growth rates and enhanced meat quality. Additionally, the development of the duodenum is intricately linked to the establishment of a robust immune system in broilers. The intestinal mucosa acts as a critical barrier against pathogens, while the microbiota plays a role in immune development [4]. Research has shown that the morphological characteristics of the duodenum, including villus height (VH) and crypt depth (CD), are indicative of its health and functionality [5]. Enhancements in these parameters correlate with improved nutrient absorption capabilities and overall health status. Furthermore, a well-developed duodenum can facilitate the rapid healing of the intestinal lining, which is essential following challenges such as viral infections or antibiotic treatments [6]. Stressors affecting the duodenum, such as the quality of feed and environmental conditions, can lead to poor development and subsequent health issues in broilers, including enteritis and dysbiosis. These health challenges ultimately translate to decreased feed efficiency, increased medication costs, and reduced production yields. Thus, understanding the developmental biology of the duodenum in broilers is essential for optimizing production practices and improving the health and welfare of poultry.

Histone phosphorylation is a significant epigenetic modification that influences chromatin structure and gene expression in various biological processes, including intestinal development [7]. This post-translational modification serves as a regulatory mechanism for transcriptional activation or repression, thus playing crucial roles in cellular differentiation, proliferation, and response to environmental stimuli. In the context of intestinal development, histone phosphorylation can modulate key developmental genes involved in epithelial cell differentiation and function. For example, specific phosphorylated histones have been shown to correlate with the expression of genes involved in intestinal barrier function, goblet cell differentiation, and enterocyte maturation, thereby critically influencing the overall architecture and function of the intestinal epithelium [8]. Additionally, histone phosphorylation enhances cellular defense mechanisms against pathogens by regulating the expression of immune-related genes [9]. The intestinal epithelium acts as a frontline defense, and its ability to respond to infections relies on the dynamic regulation of gene expression mediated by histone modifications [10]. Histone phosphorylation is particularly important for the activation of inflammatory responses and production of antimicrobial peptides, which protect the intestinal mucosa from bacterial infections [11]. Furthermore, studies have demonstrated that alterations in histone phosphorylation patterns can lead to abnormal intestinal development and increased susceptibility to diseases, emphasizing the significance of this epigenetic modification in maintaining intestinal health [12]. Given the diverse roles of histone phosphorylation in regulating gene expression during intestinal development, understanding these processes can provide insights into the mechanisms that govern gut health and disease resistance in livestock.

Despite the known significance of histone phosphorylation in various biological contexts, its specific role in the development of the broiler duodenum remains largely unexplored. This gap in knowledge calls for a systematic examination of the phosphorylation events associated with critical genes during duodenal development in broilers. The primary objective of this study is to screen for key genes associated with histone phosphorylation in the early development of the broiler duodenum. By employing transcriptomic and morphological analyses, we aim to uncover histone phosphorylation-associated genes and its effects on intestinal development. This research will provide a foundational understanding of how histone modifications contribute to the health and growth of broilers, ultimately offering valuable insights for improving broiler welfare and production practices.

## MATERIALS AND METHODS

### Animal and diet

Ten healthy Ross broilers at hatch, purchased from the Bengbu Dacheng Food. breeder farm, were selected at hatch and reared in a controlled environment optimized for growth and welfare. During the first week, the temperature was maintained between 32°C–34°C, with gradual reductions of 2°C per week until a stable temperature of 24°C was reached by week three. Humidity levels were kept at approximately 60%–70% to prevent respiratory stress and ensure optimal feed and water consumption. Lighting was managed on a 24-hour cycle, with 18 hours of light and 6 hours of darkness in the initial week to facilitate feed intake and reduce stress. This light regimen was gradually adjusted to a standard 23:1 light-to-dark cycle by the end of the first week, promoting healthy development. Clean, fresh water was provided *ad libitum* throughout the rearing period. The specialized broiler diet used in this study was sourced from Anhui Baixin Feed and formulated according to the Nutrient Requirements of Poultry [13], as detailed in Table 1. This nutritionally complete diet met the broilers’ requirements for energy, protein, amino acids, and essential minerals. Standard vaccination protocols were strictly implemented throughout the rearing period, and daily monitoring of water intake and feed consumption was conducted to ensure compliance with welfare guidelines.

### Duodenal collection and morphological analysis

At hatch (D0) and 7 days post-hatch (D7), five animals per period were humanely euthanized using 150 mg/kg sodium pentobarbital via intraperitoneal injection, in accordance with the Guidelines for Euthanasia of Laboratory Animals of China (GB/T 39760-2021), respectively. Duodenal samples were then collected for morphological analysis. The samples were fixed in 10% formalin and processed for histological examination. After dehydration and embedding in paraffin, 5 μm thick sections were cut using a microtome. These sections were stained with hematoxylin and eosin (H&E) to visualize the tissue architecture. Morphometric measurements were conducted to assess duodenal morphology, specifically the duodenal VH, CD, and the VH/CD ratio. Measurements were performed using a calibrated micrometer under a light microscope. The VH was defined as the distance from the tip of the villus to the base of the crypt, while the CD was measured from the base of the crypt to the muscularis mucosae. Statistical analyses were conducted using IBM SPSS Statistics (Version 22; IBM). Morphometric differences in duodenal VH, CD, and VH/CD ratio between the D7 and D0 groups were evaluated using an independent samples t-test, with statistical significance set at p<0.05 (two-tailed). Normality and homogeneity of variances were confirmed using the Shapiro-Wilk and Levene’s tests, respectively, prior to performing parametric analyses.

### RNA isolation and microarray quality control procedures

Total RNA was extracted from duodenal tissue using TRIzol (Invitrogen) according to manufacturer’s protocol. To ensure representative sampling of total duodenal RNA, 12–15 biological replicates from two time points (D0 and D7) were pooled to create three biological composite samples for each time point, each comprising 4–5 individual RNA specimens. RNA quality was confirmed by Agilent 2100 BioAnalyzer (RIN>8.0) and spectrophotometry (A260/A280 = 1.8–2.0). For microarray analysis, 2 μg RNA was reverse transcribed using T7-oligo (dT)24 primers (Invitrogen) with RNase H treatment. dsDNA was synthesized via DNA Polymerase I/Ligase and phenol-purified. Biotin-cRNA was produced using Affymetrix IVT Kit (≥20 μg yield), then fragmented (94°C, 35 min) in Tris-acetate/KOAc/MgOAc buffer (pH 8.1). Hybridization mixtures contained: 15 μg fragmented cRNA and MES-based buffer with spike-in controls (BioB/C/D, Cre). Arrays were hybridized (45°C, 16 h, 60 rpm), washed (stringent: 50°C/6×SSPE; non-stringent: 25°C), and stained with streptavidin-PE/anti-streptavidin. Signals were captured by GeneChip Scanner 3000 (570 nm, 3 μm). Data were processed using GCOS 1.1 (MAS 5.0 algorithm, median target = 500). All data (GSE15413) and platform details (GPL3213) are MIAME-compliant in NCBI GEO.

### Transcriptional data collection

This study utilized the gene expression microarray dataset GSE15413, obtained from the GEO database (https://www.ncbi.nlm.nih.gov/geo/). This dataset consists of six chicken duodenum samples: three from D0 (GSM386843-845) and three from D7 (GSM386846-848). In addition, histone phosphorylation-related genes were obtained from the GeneCards database (https://www.genecards.org/).

### Histone phosphorylation-related differentially expressed genes

Differentially expressed genes (DEG) analyses were conducted using GEO2R (http://www.ncbi.nlm.nih.gov/geo/geo2r). This tool employs the Limma package, an R-based software specifically designed for microarray data analysis. The Limma package offers various methods for p-value adjustment, with the default being the Benjamini & Hochberg false discovery rate technique. Genes with |log2 fold change (FC)|≥1.00 and p<0.05 were classified as DEGs. Probes without Entrez gene annotations were excluded from the analysis. Subsequently, the common subset of total DEGs and histone phosphorylation-related genes was identified as histone phosphorylation-related DEGs.

### Functional enrichment analysis and protein classification

To explore the functional roles of the DEGs, we conducted Gene Ontology (GO), KEGG, and Reactome enrichment analyses using the DAVID database (https://david.ncifcrf.gov/summary.jsp). Additionally, functional enrichment analysis was performed with the PANTHER database (http://pantherdb.org/tools/compareToRefList.jsp). To identify proteins associated with the DEGs, we classified these proteins using the PANTHER database.

### Protein-protein interaction networks, hub genes and their functions

Protein-protein interaction (PPI) networks based on the DEGs were constructed using STRING software (https://string-db.org/) and visualized with Cytoscape 3.8.0. Hub genes were identified using the CytoHubba plugin in Cytoscape, employing four different methods: MCON, DNMC, EPC, and MNC. The genes identified by these methods were designated as hub genes. Subsequently, the functions of these hub genes were summarized using information from existing literature, the NCBI database, and GeneCards.

## RESULTS AND DISCUSION

### Duodenal histomorphology in the early development

**Figure 1:**
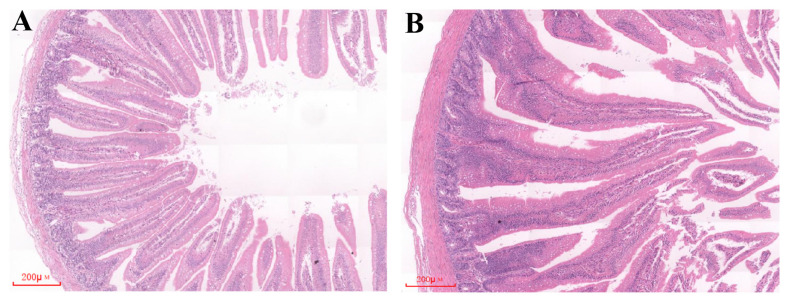
Histomorphological changes in the duodenum during early post-hatching development. (A) and (B) depict duodenal histomorphology at D0 and D7 post-hatching, respectively. On the day of hatch (D0) and 7 days post-hatch (D7), five broiler chickens per time point were humanely euthanized by intraperitoneal injection of sodium pentobarbital (150 mg/kg), following the Guidelines for Euthanasia of Laboratory Animals of China (GB/T 39760-2021). Duodenal segments were subsequently collected for morphological assessment. The tissue samples were fixed in 10% neutral buffered formalin and processed for histological analysis. Following dehydration and paraffin embedding, serial sections of 5 μm thickness were prepared using a microtome. The sections were stained with hematoxylin and eosin (H&E) for microscopic evaluation of tissue morphologyCompared with the D0, the D7 exhibited a 63.02% increase in VH and a 17.81% reduction in CD. Consequently, the VH/CD ratio demonstrated a 100.68% elevation, indicating enhanced intestinal absorptive efficiency. VH, villus height; CD, crypt depth.

As shown in Table 2 and Figure 1, we observed significant changes in the histomorphology of duodenum during the first week after hatching in broilers. In brief, compared to D0, the VH increased significantly by 63.02% on D7, while the CD decreased by 17.81%. Additionally, the VH/CD ratio significantly increased by 100.68%.

### Overview of genes expression in chicken duodenums

A total of 38,535 transcripts (Supplement 1), 4,088 differentially expressed transcripts (Supplement 2), and 12,633 genes (Supplement 3) were identified in chicken duodenum samples at D0 and D7. Additionally, 2,292 DEGs were obtained in chicken duodenum between D0 and D7, in which 1,052 and 1,240 genes were significantly up- and down-regulated in duodenum at D7 compared to D0, respectively (Supplement 4 and Supplement 5 [Table S1]). Supplement 5 (Tables S2, S3) presented the top 30 significantly up- and down-regulated genes in the duodenum at D7 compared to those D0. These upregulated genes included *AVD*, *B2M*, *BF2*, and *CDO1*, et al (Supplement 5 [Table S2]). Down-regulated genes included *ATP12A*, *FET1*, and *ACSM5*, et al (Supplement 5 [Table S3]).

**Figure 2:**
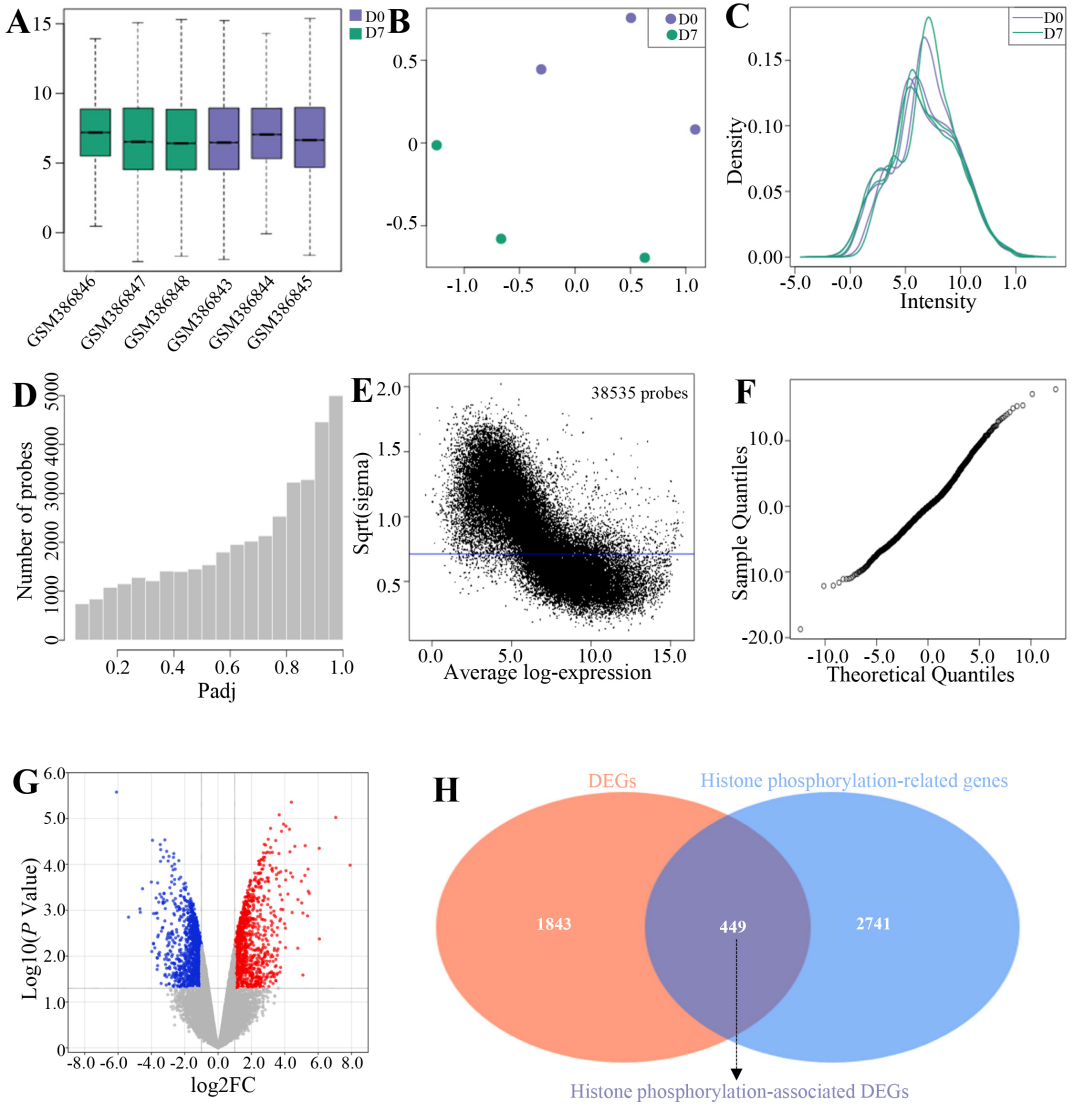
Overview of genes expression in duodenum. (A) illustrate the distribution of gene expression through a box plot. (B) present UMAP analysis of the data. (C) illustrate the expression density. (D) display adjusted p-value counts. (E) illustrate the mean-variance trends. (F) show the moderated t statistics. (G) present volcano plots of DEGs. (H) display histone phosphorylation-associated DEGs identified in duodenum. DEG, differentially expressed genes.

Figures 2A–2F indicated gene expression box plots, UMAP, expression density, adjusted p-value counts, mean-variance trend, transcript expression, moderated T statistic. Figure 2G revealed the volcano plot for the DEGs in chicken duodenum between D7 and D0. Importantly, 449 histone phosphorylation-related DEGs were obtained, in which 163 and 286 genes were significantly up- and down-regulated in duodenum at D7 compared to D0, respectively (Supplement 5 [Table S4], Figure 2H, and Supplement 6). Supplement 5 (Tables S5, S6) illustrated the top 30 significantly up- and down-regulated histone phosphorylation-related DEGs in the duodenum at D7 compared to those D0. These upregulated genes included *P2RX7*, *IL1B*, *SORBS2*, and *SUCLG1*, et al (Supplement 5 [Table S5]). Down-regulated genes included *SCRIB*, *CIT*, *CBX7*, and *ELK4*, et al (Supplement 5 [Table S6]).

### GO enrichment

**Figure 3:**
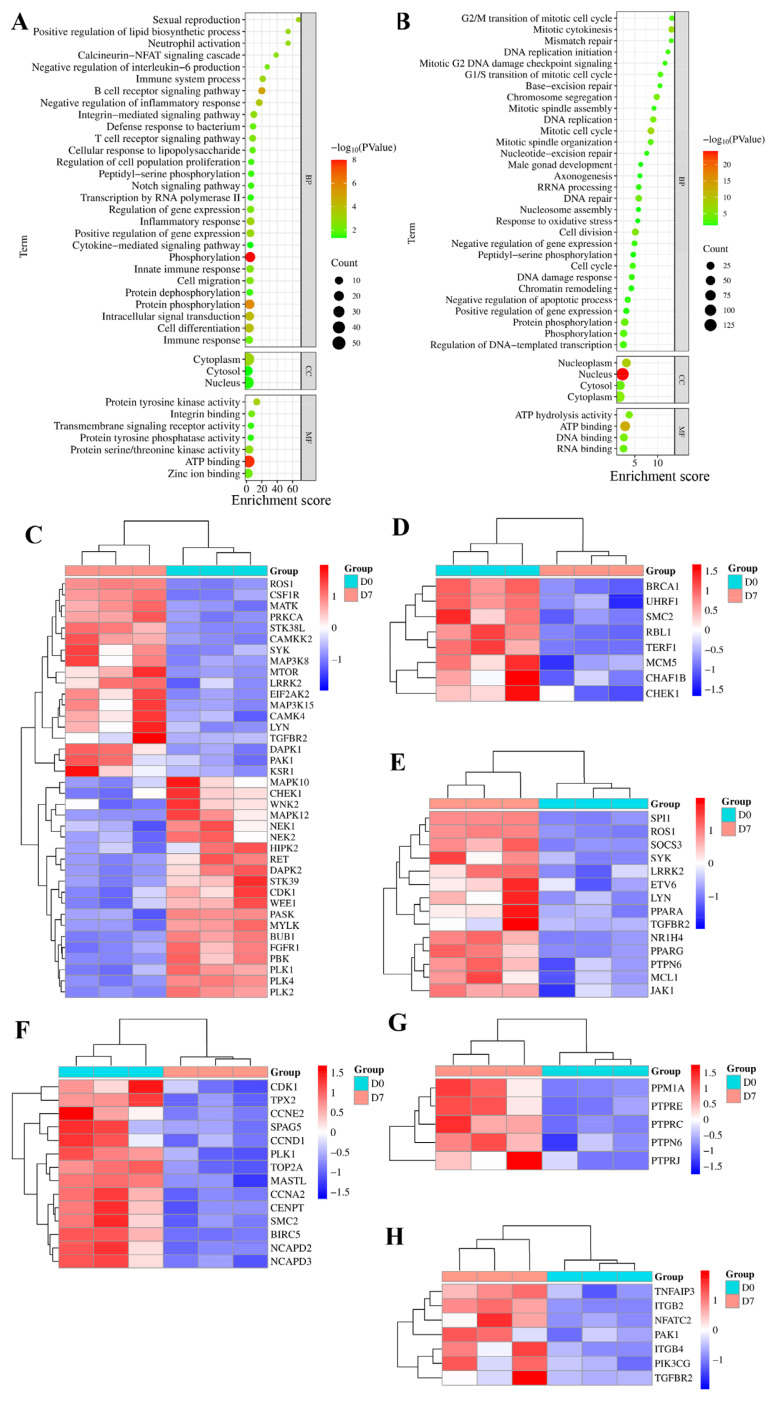
Functional enrichment of histone phosphorylation-associated DEGs. (A) GO terms enriched for upregulated histone phosphorylation-linked DEGs in the duodenum at D7, including phosphorylation (e.g., protein phosphorylation/dephosphorylation), intracellular signal transduction, and cell differentiation. (B) Enriched GO terms for downregulated histone phosphorylation-related DEGs, predominantly involving chromatin remodeling, cell division, and phosphorylation-associated regulatory processes. (C–H) illustrate the histone phosphorylation-related genes in the biological processes of protein phosphorylation, cell cycle, cell differentiation, cell division, protein dephosphorylation and cell migration, respectively. DEG, differentially expressed genes.

To understand the biological processes associated with differential histone phosphorylation-related genes, GO enrichment analysis was conducted using the DAVID database. Figure 3A and Supplement 7 illustrated the biological processes linked to the up-regulated histone phosphorylation-related genes in chicken duodenum at D7. These processes included phosphorylation, protein phosphorylation, intracellular signal transduction, cell differentiation, protein dephosphorylation, inflammatory response, immune response, and cell migration. Furthermore, down-regulated histone phosphorylation-related genes were associated with biological processes, including protein phosphorylation, chromatin remodeling, cell division, phosphorylation, mitotic cell cycle, mitotic cytokinesis, chromosome segregation, and cell cycle (Figure 3B and Supplement 8). Additionally, Figures 3C–3H illustrated the histone phosphorylation-related genes in the biological processes of protein phosphorylation, cell cycle, cell differentiation, cell division, protein dephosphorylation and cell migration, respectively.

### KEGG enrichment

**Figure 4:**
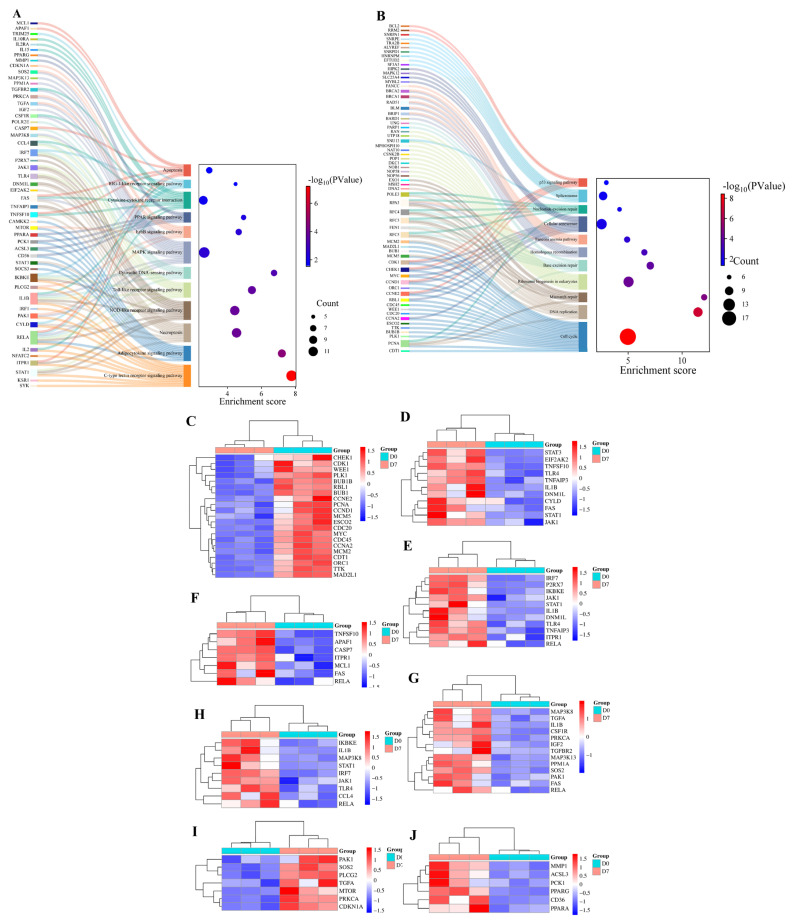
Pathway enrichment analysis of histone phosphorylation-associated DEGs. (A) KEGG pathways enriched for upregulated histone phosphorylation-linked DEGs in the duodenum at D7, highlighting immune and metabolic regulation (e.g., MAPK, ErbB, PPAR, Toll-like receptor, and necroptosis pathways) alongside stress-responsive processes (C-type lectin receptor, NOD-like receptor, and adipocytokine signaling). (B) Pathways associated with downregulated histone phosphorylation-related DEGs, predominantly involving genomic stability mechanisms (p53 signaling, mismatch/base excision/nucleotide excision repair, homologous recombination) and cell cycle regulation (spliceosome, DNA replication, cellular senescence). (C–J) present the histone phosphorylation-related genes in the signaling pathways of cell cycle, necroptosis, NOD-like receptor, apoptosis, MAPK, ErbB, and PPAR respectively. DEG, differentially expressed genes.

To understand the signaling pathways associated with differential histone phosphorylation-related genes, KEGG enrichment analysis was conducted using the DAVID database. Figure 4A and Supplement 9 illustrated the signaling pathways linked to the up-regulated histone phosphorylation-related genes in chicken duodenum at D7. These signaling pathways included MAPK, ErbB, PPAR, apoptosis, C-type lectin receptor, NOD-like receptor, adipocytokine, necroptosis, Toll-like receptor, cytosolic DNA-sensing, cytokine-cytokine receptor interaction, and RIG-I-like receptor. Furthermore, the down-regulated histone phosphorylation-related genes in chicken duodenum at D7. These signaling pathways included p53, spliceosome, cell cycle, mismatch repair, base excision repair, homologous recombination, DNA replication, cellular senescence, and nucleotide excision repair (Figure 4B and Supplement 10). Additionally, Figures 4C–4J illustrated the histone phosphorylation-related genes in the signaling pathways of cell cycle, necroptosis, NOD-like receptor, apoptosis, MAPK, ErbB, and PPAR respectively.

### Reactome pathway analysis

**Figure 5:**
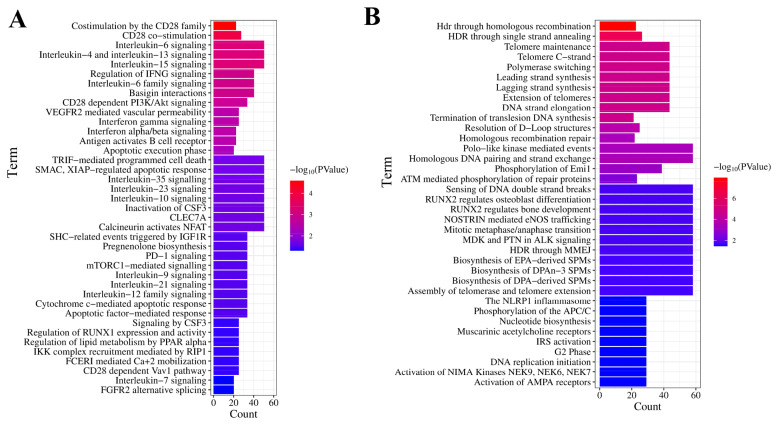
Reactome pathway enrichment of histone phosphorylation-associated DEGs. (A) Upregulated histone phosphorylation-associated DEGs in D7 duodenum were enriched in metabolic regulation (pregnenolone biosynthesis), cell survival signaling (CD28-dependent PI3K/Akt, mTORC1), and apoptosis. (B) Downregulated histone phosphorylation-associated DEGs primarily involved DNA replication fidelity (polymerase switching, lagging strand synthesis, replication initiation), telomere dynamics (maintenance, C-strand extension), and mitotic control (APC/C phosphorylation, metaphase-anaphase transition). DEG, differentially expressed genes.

Figure 5A illustrated the signaling pathways linked to the up-regulated histone phosphorylation-related genes in chicken duodenum at D7. The up-regulated histone phosphorylation-related genes in chicken duodenum at D7 played an important role in CD28 dependent PI3K/Akt signaling, apoptotic factor-mediated response, mTORC1-mediated signaling, and pregnenolone biosynthesis, et al. Additionally, the down-regulated histone phosphorylation-related genes at D7 involved in APC/C phosphorylation, mitotic metaphase/anaphase transition, polymerase switching, telomere maintenance, telomere C-strand, telomeres extension, DNA strand elongation, lagging strand synthesis, DNA replication initiation, and (Figure 5B).

### Protein classification

**Figure 6:**
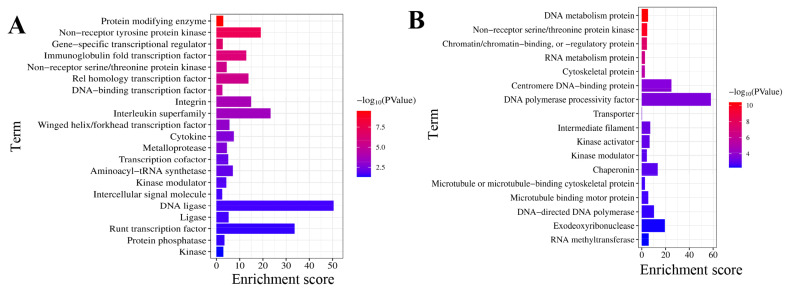
Functional classification of histone phosphorylation-associated DEGs. (A) Upregulated DEGs at D7 encoded proteins critical for immune signaling (interleukin superfamily, cytokines), molecular connectivity (integrins, intercellular signal molecules), and biosynthesis (aminoacyl-tRNA synthetases, DNA ligases). (B) Downregulated DEGs functionally centered on genomic maintenance (DNA helicases, polymerase processivity factors), proteostasis (HSP90 chaperones, general chaperones), and RNA processing machinery. DEG, differentially expressed genes.

To understand the proteins associated with differential histone phosphorylation-related genes, Protein classification analysis was conducted using the PANTHER database. The upregulated histone phosphorylation-related genes at D7 were associated with DNA ligase, interleukin superfamily, integrin, cytokine, aminoacyl-tRNA synthetase, ligase, and intercellular signal molecules, et al (Figure 6A). Additionally, the down-regulated histone phosphorylation-related genes in chicken duodenum at D7 were linked to RNA processing factor, cytoskeletal protein, chaperone, kinase activator, DNA helicase, HSP90 family chaperone, and DNA polymerase processivity factor, et al (Figure 6B).

### Protein-protein interaction network, hub genes and their functions

**Figure 7:**
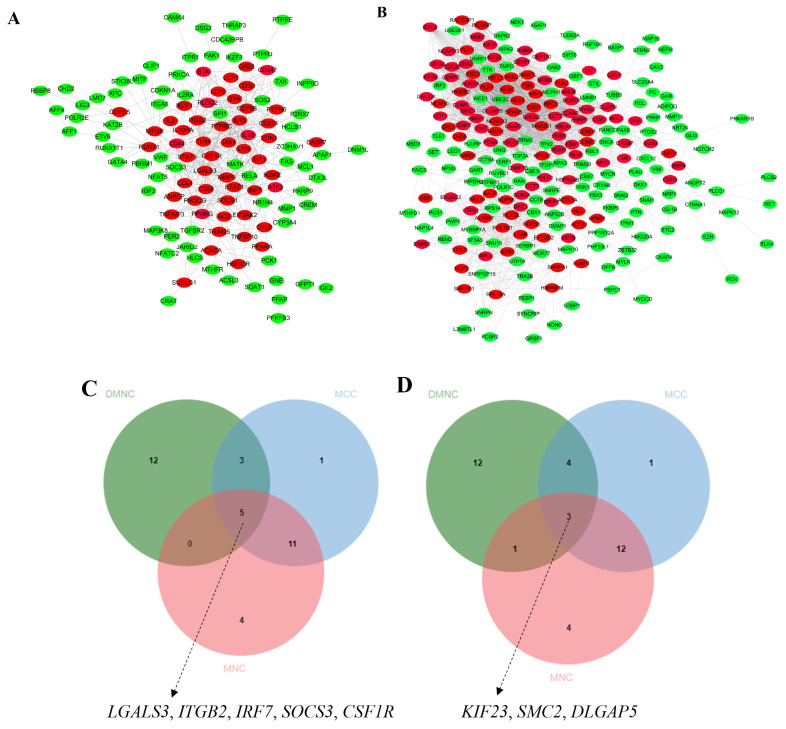
Protein interaction networks and hub genes regulating duodenal development. (A,B) PPI network analysis identified upregulated immune regulators (*TLR4*, *IL2/15*, *CSF1R*, *PPARG*) and cell adhesion mediators (*CD44* and *ITGB2*) alongside downregulated cell cycle controllers (*CCND1* and *CCNE2*) and chromosomal stability factors (*SMC2* and *KIF23*) in duodenal development. (C,D) Hub gene prioritization refined key players: upregulated *LGALS3* (glycan-mediated signaling), *SOCS3* (inflammatory regulation), and *IRF7* (innate immunity), contrasted with downregulated mitotic drivers (*KIF23* and *DLGAP5*) and chromatin organizers (*SMC2*). PPI, protein-protein interaction.

To further identify key genes, we performed a PPI analysis of the DEGs using STRING. As depicted in Figure 7A, among the upregulated histone phosphorylation genes, many genes, including *TLR4*, *LGALS3*, *CD44*, *ITGB2*, *CCL4*, *SOCS3*, *IL2*, *JAK1*, *IL15*, *CSF1R*, and *PPARG* were implicated in the early development of the broiler duodenum. Additionally, within the downregulated histone phosphorylation genes, many genes such as *CCND1*, *KIF23*, *DLGAP5*, *CCNE2*, *SMC2*, *BMP4*, and *CAV1* were also associated with the early development of the broiler duodenum (Figure 7B).

To further identify hub genes, we analyzed the PPI network using the CytoHubba plugin in Cytoscape, employing three algorithms: DMNC, MCC, and MNC. The common genes identified by these three algorithms were designated as hub genes. As illustrated in Figure 7C, among the upregulated histone phosphorylation genes, *LGALS3*, *ITGB2*, *IRF7*, *SOCS3*, and *CSF1R* were associated with the early development of the broiler duodenum. Additionally, in the downregulated histone phosphorylation genes, *KIF23*, *SMC2*, and *DLGAP5* were found to be involved in the early development of the broiler duodenum (Figure 7D). In addition, hub gene functions were shown in Table 3.

## DISCUSSION

Histone phosphorylation dynamically regulates gene expression through bidirectional mechanisms: 1) Activation occurs when phosphorylation neutralizes histone-DNA electrostatic interactions, destabilizing nucleosomes to permit transcriptional machinery access while simultaneously recruiting scaffold proteins that bridge activators to basal transcription factors, as demonstrated during immediate-early gene induction; 2) Repression arises when phosphorylated histones serve as docking sites for silencing complexes like trigger adjacent residue modifications (methylation/acetylation crosstalk), with the phosphorylated C-terminal domain of RNA polymerase II further modulating elongation efficiency, creating a phosphorylation-dependent “histone code” that integrates cellular signaling with chromatin state transitions across mitosis, DNA repair, and stress responses [14–17]. Prior studies have demonstrated that histone phosphorylation critically regulates gene expression and chromatin dynamics during early intestinal morphogenesis [18]. In the duodenum, this modification orchestrates epithelial cell proliferation, differentiation, and survival—processes fundamental to establishing gut integrity, nutrient absorption efficiency, and immune competency [19]. Broilers exhibit heightened dependence on early duodenal development, as these structural adaptations directly determine metabolic performance and productivity. Our findings reveal substantial morphological remodeling during this phase, including villus elongation (height increase) and optimized villus-to-crypt ratios—key adaptations for expanding absorptive surface area [20]. Notably, as broilers age (from D0 to D7), the intestinal VH increases accordingly, indicating a positive balance between intestinal cell proliferation and apoptosis (i.e., cell proliferation predominates over apoptosis). Through transcriptional network analysis, we identified eight histone phosphorylation-associated hub genes (*LGALS3, ITGB2*, *IRF7*, *SOCS3*, *CSF1R*, *KIF23*, *SMC2*, and *DLGAP5*) that mechanistically link epigenetic regulation to developmental programming.

*LGALS3* (galectin-3) plays critical roles in macrophage chemotaxis, mucosal barrier maintenance, intestinal epithelial cell (IEC) apoptosis regulation, and inflammatory responses [21–23]. Our study revealed a 6.59-fold increase in *LGALS3* gene expression in the duodenum at D7 compared to D0 (Supplement 6), with functional analysis confirming its involvement in macrophage chemotaxis (Supplement 7). These findings align with Sun et al [21], who reported that *LGALS3* silencing in necrotizing enterocolitis models inhibited the TLR4/NF-κB pathway, subsequently reducing IEC apoptosis and inflammation [21]. Emerging evidence further suggests LGALS3’s protective functions through ER stress modulation, autophagy regulation, and inflammasome control in intestinal Behçet’s disease [22], along with its capacity to upregulate key mucosal barrier components (MUC2, Occludin, and ZO-1) [23]. Collectively, these observations suggest *LGALS3* promotes duodenal development through: 1) mucosal barrier reinforcement via tight junction protein upregulation, 2) inflammatory control through TLR4/NF-κB-mediated macrophage regulation, and 3) cellular homeostasis maintenance via ER stress/autophagy pathways.

*ITGB2* (Integrin beta 2) plays a pivotal role in maintaining gut epithelial homeostasis through its involvement in cell adhesion, regulation of intestinal inflammation, modulation of permeability, and mediation of immune responses. In murine models of inflammatory bowel disease (IBD), elevated *ITGB2* expression has been observed, while its suppression exacerbates disease pathology [24]. Recent findings by Wang et al [25] further elucidate *ITGB2*’s regulatory functions in immune response modulation and neuroinflammatory processes associated with intestinal disorders [26]. Complementing these observations, Zeng et al [26] established that *ITGB2* is essential for maintaining cytoskeletal integrity and facilitating cell adhesion, with its activation stimulating cellular proliferation and conferring protection against LPS-induced apoptosis in intestinal cells [26]. Our experimental data revealed a 2.81-fold increase in *ITGB2* gene expression in the duodenum between D0 and D7 (Supplement 6). Functional analysis identified *ITGB2*’s association with critical biological processes including cell migration, integrin-mediated signaling cascades, and integrin-dependent cell adhesion mechanisms (Supplement 7). Collectively, these findings support our hypothesis that *ITGB2* serves as a key regulator in the early developmental stages of the broiler duodenum, orchestrating multiple physiological processes such as cellular adhesion, migratory behavior, inflammatory responses, barrier function maintenance, and immune system modulation.

*IRF7* (Interferon regulatory factor 7) serves as a master transcriptional regulator orchestrating critical aspects of intestinal homeostasis, including inflammatory responses, immune surveillance, epithelial barrier integrity, and macrophage polarization dynamics. Our experimental data revealed a striking 3.98-fold upregulation of *IRF7* gene expression in the duodenum between D0 and D7 (Supplement 6), suggesting its potential involvement in early intestinal development. Functional annotation further associated *IRF7* with essential biological processes such as immune system regulation, hematopoietic development (Supplement 7), and Toll-like receptor signaling cascades (Supplement 9), corroborating previous findings by Qing et al [27]. The pivotal role of *IRF7* in intestinal pathophysiology was substantiated by Qing et al [27], who documented significantly diminished *IRF7* expression in ulcerative colitis patients [27]. Their mechanistic studies using *IRF7*-deficient murine models demonstrated exacerbated susceptibility to DSS-induced colitis, characterized by heightened systemic and colonic pro-inflammatory cytokine profiles. This pathological phenotype was mechanistically linked to disrupted intestinal barrier function, evidenced by dysregulated expression of critical junctional proteins (β-catenin, Occludin, E-cadherin) and the mucin barrier component MUC2, along with reduced interleukin-28A (IL-28A) levels. Importantly, their work established that IL-28A treatment could functionally rescue barrier defects by upregulating these key epithelial integrity markers [27].

Suppressor of cytokine signaling 3 (*SOCS3*) functions as a critical negative feedback regulator in cytokine signaling pathways, playing an indispensable role in maintaining immune homeostasis by preventing hyperactive cellular responses. Emerging evidence has implicated epigenetic modifications of *SOCS3*, particularly promoter methylation in colonic mucosa, as a molecular hallmark of the inflammatory pathology characteristic of Crohn’s Disease [28]. Beyond its regulatory functions, *SOCS3* exhibits multifaceted protective roles in intestinal physiology: it promotes the polarization of M2 anti-inflammatory macrophages and confers protection against intestinal ischemia/reperfusion injury [29]. At the molecular level, *SOCS3* precisely modulates IL-22 responsiveness in colonic epithelial cells [30], while simultaneously orchestrating the upregulation of crucial junctional proteins (ZO-1, occludin, and E-cadherin) that constitute the intestinal epithelial barrier. These molecular mechanisms collectively underlie *SOCS3*’s demonstrated capacity to ameliorate inflammatory processes and restore epithelial integrity in experimental models of DSS-induced colitis [31].

Colony-stimulating factor 1 receptor (*CSF1R*) emerges as a pivotal regulator in intestinal development and homeostasis, with our study revealing a 1.58-fold increase in its duodenal expression between D0 and D7 (Supplement 6). This developmental upregulation coincides with CSF1R’s involvement in critical signaling pathways, including protein phosphorylation (Supplement 7), MAPK cascade, and cytokine-cytokine receptor interactions (Supplement 9), suggesting its potential role in orchestrating intestinal maturation [32,33]. While best known for mediating oxidative stress-induced premature senescence and inflammatory responses, *CSF1R* exhibits dual functionality in gut physiology. On one hand, its inhibition attenuates Microcystin-LR-induced colorectal inflammation [34]; on the other, it participates in a pathological triad of chronic inflammation, fibrosis, and barrier dysfunction that disrupts gut microbiota equilibrium and metabolite profiles [35]. Importantly, *CSF1R*’s developmental expression pattern may reflect its balanced regulation of pro-inflammatory and tissue-repair mechanisms during intestinal morphogenesis. The observed temporal expression dynamics of *CSF1R* in developing duodenum, coupled with its established roles in mucosal immunity and barrier maintenance, position this receptor as a potential modulator of intestinal developmental programming. Its capacity to influence both inflammatory cascades and epithelial integrity suggests *CSF1R* may serve as a molecular switch coordinating immune maturation with structural development in the nascent gut.

Kinesin family member 23 (*KIF23*), a critical regulator of mitotic spindle midzone assembly [36], demonstrated significant developmental regulation in our study. We observed a 54% reduction in duodenal *KIF23* expression at D7 compared to D0 (Supplement 6), paralleling its functional association with cell division machinery (Supplement 8). This developmental downregulation aligns with established evidence that *KIF23* overexpression induces duodenal pathology, including crypt reduction [36], impaired cell cycle progression, and suppressed mitosis-related gene expression (*ASPM*, *CCNB1*, and *BIRC5*) [36]. Our findings suggest that controlled *KIF23* suppression during intestinal development may optimize broiler epithelial proliferation and differentiation through mitotic regulation. Structural maintenance of chromosomes 2 (*SMC2*), essential for chromosomal cohesion and segregation, showed a 60.77% expression decrease in developing duodenum (Supplement 6). Its developmental regulation correlates with critical mitotic processes including chromosome condensation and segregation (Supplement 8) [37,38], reinforcing its role in maintaining genomic stability during rapid intestinal growth.

The coordinated developmental regulation of these mitotic regulators extends to *DLGAP5*, which exhibited a 62.37% expression reduction at D7 (Supplement 6). Like *KIF23*, *SMC2*, and *DLGAP5* participates in chromosome segregation and kinetochore assembly (Supplement 8), with its dosage sensitivity influencing both intestinal homeostasis and pathology [39–42]. The parallel downregulation of these three mitotic regulators (*KIF23*, *SMC2*, *DLGAP5*) suggests an evolutionarily conserved mechanism modulating cell division precision during intestinal morphogenesis.

While this study identified several hub genes associated with broiler duodenal development through integrated morphological and transcriptional analyses, the following limitations should be acknowledged: (1) Lack of experimental validation for hub gene expression patterns. Although bioinformatics tools and co-expression networks revealed key candidate genes, their spatial and temporal expression profiles in the duodenum remain unverified. Experimental approaches such as quantitative PCR, in situ hybridization, or immunohistochemistry are critical to confirm their localization and dynamic expression across developmental stages. Without such validation, the reliability of transcriptional data and their biological relevance to duodenal morphogenesis cannot be fully established. (2) Uncharacterized functional roles and mechanistic insights. The study primarily focused on gene identification and network analysis but did not explore the functional contributions of these hub genes to duodenal development. Future work should employ loss-of-function or gain-of-function experiments (e.g., CRISPR/Cas9 knockout, RNA interference, or overexpression models) to assess their impact on critical processes like cell proliferation, apoptosis, or stem cell maintenance. Additionally, the mechanistic links between histone phosphorylation and duodenal development require clarification. These unresolved questions highlight the need for multi-omics integration (e.g., ChIP-seq for histone modifications, proteomics) and in vivo functional assays to bridge the gap between gene expression patterns and biological mechanisms. Addressing these limitations will strengthen the translational relevance of the findings for poultry science and developmental biology.

## CONCLUSION

This study demonstrates histone phosphorylation as an epigenetic orchestrator of duodenal maturation in broilers, driving villus expansion and crypt remodeling to enhance absorptive capacity. Transcriptomic analysis identified 449 phosphorylation-regulated DEGs enriched in MAPK/PPAR signaling and immune-cell cycle integration. Eight hub genes (*LGALS3*, *ITGB2*, *IRF7*, *SOCS3*, *CSF1R*, *KIF23*, *SMC2*, *DLGAP5*) collectively bridge mucosal specialization, metabolic adaptation, and mitotic precision, offering novel targets for optimizing poultry gut development through epigenetic modulation.

## Figures and Tables

**Table 1:** Dietary composition and nutrient level

Item	Content, %
Ingredients	
Corn	41.5
Rice polishing meal	8.8
Soybean meal	23.6
Wheat	8.0
Rice bran	5.0
Corn starch residue	3.0
Spray-dried corn gluten feed	2.0
Soybean meal	3.2
Feather meal	1.0
Limestone	1.8
Dicalcium phosphate	0.5
Montmorillonite	0.3
Sodium chloride	0.3
Lysine	0.49
Methionine	0.24
Threonine	0.02
Choline chloride	0.1
VTR Enzyme 818	0.03
Phytase	0.02
Sodium bicarbonate	0.1
Total	100
Nutrients levels^1)^	
Metabolizable energy/Mcal·kg^−1^	2.90
Moisture	9.74
Crude protein	19.22
Crude fat	3.07
Crude fiber	5.38
Crude ash	10.05
Lysine	1.35
Methionine	0.60
Methionine+cystine	0.80
Calcium	0.88
Available phosphorus	0.40

1) Content of metabolizable energy, methionine, cystine, calcium, available phosphorus in diet was calculated values, while moisture, crude protein, fat, fiber, ash was measured values.

**Table 2:** Duodenal tissue morphology of broilers at D0 and D7

Items	D0	D7
Villus heigh/μm	749.58±103.99^b^	1,221.84±68.93^a^
Crypt depth/μm	63.42±3.72^a^	52.12±6.55^b^
Villus height/crypt depth	11.81±1.43^b^	23.70±2.91^a^

a,b Values in the same row sharing the same lowercase letter superscript or no superscript indicate no significant difference (p>0.05), whereas distinct lowercase letter superscripts denote significant differences (p<0.05).

**Table 3:** Hub genes and their functions

Gene symbol	Full name	Functions
*LGALS3*	Galectin 3	*LGALS3* encodes a member of the galectin family of carbohydrate binding proteins which play an important role in numerous cellular functions including apoptosis, innate immunity, cell adhesion and T-cell regulation. The protein exhibits antimicrobial activity against bacteria and fungi.
*ITGB2*	Integrin subunit beta 2	*ITGB2* encodes an integrin beta chain, which plays an important role in immune response and defects in this gene cause leukocyte adhesion deficiency.
*IRF7*	Interferon regulatory factor 7	*IRF7* encodes interferon regulatory factor 7, a member of the interferon regulatory transcription factor (IRF) family. It plays a critical role in the innate immune response against DNA and RNA viruses and regulates the transcription of type I IFN genes and IFN-stimulated genes by binding to an interferon-stimulated response element in their promoters.
*SOCS3*	Suppressor of cytokine signaling 3	*SOCS3* encodes a member of the STAT-induced STAT inhibitor (SSI), also known as suppressor of cytokine signaling (SOCS), family. SSI family members are cytokine-inducible negative regulators of cytokine signaling. The expression of this gene is induced by various cytokines, including IL6, IL10, and interferon (IFN)-gamma.
*CSF1R*	Colony stimulating factor 1 receptor	The protein encoded by *CSF1R* is a cytokine which controls the production, differentiation, and function of macrophages. This receptor mediates most if not all of the biological effects of this cytokine.
*KIF23*	Kinesin family member 23	The protein encoded by *KIF23* is a member of kinesin-like protein family which include microtubule-dependent molecular motors that transport organelles within cells and move chromosomes during cell division.
*SMC2*	Structural maintenance of chromosomes 2	*SMC2* is predicted to enable chromatin binding activity and involved in mitotic chromosome condensation and positive regulation of chromosome condensation.
*DLGAP5*	DLG Associated Protein 5	*DLGAP5* is predicted enable microtubule binding activity and involved in several processes, including centrosome localization; kinetochore assembly; and mitotic spindle organization.

## SUPPLEMENTARY MATERIAL

**Supplement 1**. All transcripts found in the duodenum of broilers (XLSX = 3,224KB).

**Supplement 2**. Differentially expressed transcripts found in the duodenum of broilers (XLSX = 384KB).

**Supplement 3**. All genes found in the duodenum of broilers (XLSX = 1,218KB).

**Supplement 4**. DEGs found in the duodenum of broilers (XLSX = 239KB).

**Supplement 5**. Supplementary Tables.

**Supplement 6**. Histone phosphorylation-related DEGs found in the duodenum of broilers (XLSX = 55KB).

**Supplement 7**. GO enrichment analysis for upregulated histone phosphorylation-related DEGs found in the duodenum of broilers (XLSX = 20KB).

**Supplement 8**. GO enrichment analysis for downregulated histone phosphorylation-related DEGs found in the duodenum of broilers (XLSX = 25KB).

**Supplement 9**. KEGG enrichment analysis for upregulated histone phosphorylation-related DEGs found in the duodenum of broilers (XLSX = 11KB).

**Supplement 10**. KEGG enrichment analysis for downregulated histone phosphorylation-related DEGs found in the duodenum of broilers (XLSX = 11KB).
