# Supplementary material for: Morphological and transcriptional insights into the role of histone phosphorylation-related genes in early development of the chicken duodenum
Source: Anim Biosci. 2025 Jun 10;38(11):2377–91. doi: 10.5713/ab.25.0108 (PMC12580959; doi:10.5713/ab.25.0108)
Supplement: Supplementary file 5 [file ab-25-0108-supplementary-5.pdf]

Table S1. DEGs obtained in broiler duodenum between D0 and D7

Total	Up-regulated	down-regulated
2,292	1,052	1,240

Table S2. Top 30 upregulated genes identified at D7 compared to D0 Gene symbol

Log$_2$FC	*P* Value		Full name
AVD	4.36	1.54E-04	Avidin
B2M	3.84	5.93E-04	Beta-2-microglobulin
BF2	3.92	1.33E-05	Major histocompatibility complex class I antigen BF2
BHLHE22	4.04	2.46E-03	Basic helix-loop-helix family member e22
CA7	6.07	4.47E-05	Carbonic anhydrase VII
CALB1	4.07	1.47E-05	Calbindin 1, 28kDa
CD72	3.73	4.58E-04	CD72 molecule
CDO1	3.80	1.90E-05	Cysteine dioxygenase type 1
CMPK2	4.44	1.12E-03	Cytidine/uridine monophosphate kinase 2
CTSE	3.75	1.55E-03	Cathepsin E
CYP1A4	5.40	1.27E-04	Cytochrome P450 1A4
DDX60	4.28	1.72E-05	DEAD box polypeptide 60
FGG	5.09	2.59E-02	Fibrinogen gamma chain
GAL3ST2	4.31	5.33E-05	Galactose-3-O-sulfotransferase 2
GNLY	4.39	4.41E-06	Granulysin
GPRC5A	5.41	3.87E-04	G protein-coupled receptor, family C, group 5, member A
GZMA	7.06	9.47E-06	Granzyme A
IFI6	3.89	9.70E-04	Interferon, alpha-inducible protein 6
IFIT5	4.47	1.88E-04	Interferon-induced protein with tetratricopeptide repeats 5
IGLL1	5.36	1.33E-03	Immunoglobulin lambda-like polypeptide 1
IL1B	3.98	1.96E-03	Interleukin 1, beta
IL2RA	3.71	4.97E-03	Interleukin 2 receptor, alpha
INF2	3.77	9.53E-04	Inverted formin, FH2 and WH2 domain containing
JCHAIN	4.78	6.71E-03	Joining chain of multimeric IgA and IgM
KCNJ16	4.04	6.53E-03	Potassium voltage-gated channel subfamily J member 16
MST1	4.19	4.44E-04	Macrophage stimulating 1
MX1	5.21	1.74E-04	Myxovirus resistance 1, interferon-inducible protein p78
MYO1G	4.99	2.33E-04	Myosin IG
OASL	4.05	2.18E-04	2'-5'-oligoadenylate synthetase-like
P2RX7	4.07	1.37E-04	Purinergic receptor P2X 7

Table S3. Top 30 downregulated genes identified at D7 compared to D0 Gene

Log$_2$FCsymbol	*P* Value		Full name
SCRIB	-3.88	5.27E-03	Scribbled planar cell polarity protein
CIT	-3.74	1.17E-03	Citron rho-interacting serine/threonine kinase
CBX7	-3.30	6.82E-04	Chromobox homolog 7
DNASE1	-3.20	2.93E-05	Deoxyribonuclease I
RPS14	-3.14	9.35E-03	Ribosomal protein S14
AR	-2.95	6.68E-05	Androgen receptor
ELK4	-2.75	2.70E-02	ELK4, ETS-domain protein
STK39	-2.74	3.26E-03	Serine/threonine kinase 39
TRIM28	-2.68	2.56E-02	Tripartite motif containing 28
HSP90AB1	-2.66	1.18E-03	Heat shock protein 90kDa alpha, class B member 1
KRT19	-2.57	1.27E-04	Keratin 19
NEFM	-2.56	5.56E-04	Neurofilament, medium polypeptide
ZBTB16	-2.50	2.98E-02	Zinc finger and BTB domain containing 16
DAPK2	-2.44	1.02E-03	Death-associated protein kinase 2
GRIN1	-2.43	4.36E-02	Glutamate ionotropic receptor NMDA type subunit 1
INS	-2.40	8.32E-04	Insulin
SNRPG	-2.35	3.10E-02	Small nuclear ribonucleoprotein polypeptide G
PC	-2.34	1.19E-02	Pyruvate carboxylase
GTPBP3	-2.32	2.53E-02	GTP binding protein 3
GTF2H4	-2.31	4.51E-02	General transcription factor IIH, polypeptide 4, 52kDa
NOTCH2	-2.21	2.50E-02	Notch 2
CHD5	-2.19	1.28E-02	Chromodomain helicase DNA binding protein 5
KIF2C	-2.17	1.41E-03	Kinesin family member 2C
CAV1	-2.05	5.24E-04	Caveolin 1, caveolae protein, 22kDa
PASK	-2.05	9.37E-03	PAS domain containing serine/threonine kinase
DKK1	-2.04	1.41E-02	Dickkopf WNT signaling pathway inhibitor 1
GHR	-2.03	1.74E-03	Growth hormone receptor
STMN2	-2.01	9.87E-04	Stathmin-like 2
IRF2	-1.95	1.17E-02	Interferon regulatory factor 2
PAX6	-1.94	4.89E-02	Paired box 6

Table S4. Histone phosphorylation-related DEGs between D7 and D0

Total	Up-regulated	down-regulated
449	163	286

Table S5. Top 30 upregulated histone phosphorylation-related genes identified at D7 compared to D0

Gene symbol	Log_2FC	*P* Value	Full name
P2RX7	4.07	1.37E-04	Purinergic receptor P2X 7
IL1B	3.98	1.96E-03	Interleukin 1, beta
SORBS2	3.74	1.76E-02	Sorbin and SH3 domain containing 2
IL2RA	3.71	4.97E-03	Interleukin 2 receptor, alpha
NOX1	3.70	1.71E-03	NADPH oxidase 1
EDN1	3.56	2.37E-02	Endothelin 1
SUCLG1	3.37	2.86E-02	Succinate-CoA ligase, alpha subunit
CLIP1	3.34	2.58E-02	CAP-GLY domain containing linker protein 1
MCM6	3.17	1.14E-03	Minichromosome maintenance complex component 6
RUNX1	3.05	3.75E-04	Runt-related transcription factor 1
IL15	3.01	2.84E-05	Interleukin 15
CD79B	2.96	2.82E-02	CD79b molecule
MMP1	2.95	4.63E-03	Matrix metallopeptidase 1 (interstitial collagenase)
PBRM1	2.87	4.22E-03	Polybromo 1
SCG5	2.82	1.01E-02	Secretogranin V
SLC9A3	2.80	7.03E-05	Solute carrier family 9, subfamily A, member 3
ACHE	2.79	1.05E-02	Acetylcholinesterase (Yt blood group)
LGALS3	2.73	6.97E-05	Lectin, galactoside-binding, soluble, 3
VWF	2.72	2.78E-02	Von Willebrand factor
TGFBR2	2.72	1.41E-02	Transforming growth factor, beta receptor II
CD3D	2.72	5.59E-05	CD3d molecule, delta
INPP5D	2.71	5.59E-03	Inositol polyphosphate-5-phosphatase, 145kDa
NFATC2	2.62	1.57E-03	Nuclear factor of activated T-cells, cytoplasmic, calcineurin-dependent 2
RELA	2.59	3.28E-02	V-rel avian reticuloendotheliosis viral oncogene homolog A
ROS1	2.55	1.53E-04	ROS proto-oncogene 1, receptor tyrosine kinase
MMP9	2.54	3.43E-03	Matrix metallopeptidase 9
PFKFB3	2.52	7.30E-03	6-phosphofructo-2-kinase/fructose-2,6-biphosphatase 3
LMO7	2.47	1.93E-02	LIM domain 7
MITF	2.44	3.82E-02	Microphthalmia-associated transcription factor
CCL4	2.43	2.81E-02	Chemokine (C-C motif) ligand 4

Table S6. Top 30 downregulated histone phosphorylation-related genes identified at D7 compared to D0

Gene symbol	Log_2FC	*P* Value	Full name
SCRIB	-3.88	5.27E-03	Scribbled planar cell polarity protein
CIT	-3.74	1.17E-03	Citron rho-interacting serine/threonine kinase
CBX7	-3.30	6.82E-04	Chromobox homolog 7
DNASE1	-3.20	2.93E-05	Deoxyribonuclease I
RPS14	-3.14	9.35E-03	Ribosomal protein S14
AR	-2.95	6.68E-05	Androgen receptor
ELK4	-2.75	2.70E-02	ELK4, ETS-domain protein (SRF accessory protein 1)
STK39	-2.74	3.26E-03	Serine/threonine kinase 39
TRIM28	-2.68	2.56E-02	Tripartite motif containing 28
HSP90AB1	-2.66	1.18E-03	Heat shock protein 90kDa alpha, class B member 1
KRT19	-2.57	1.27E-04	Keratin 19
NEFM	-2.56	5.56E-04	Neurofilament, medium polypeptide
ZBTB16	-2.50	2.98E-02	Zinc finger and BTB domain containing 16
DAPK2	-2.44	1.02E-03	Death-associated protein kinase 2
GRIN1	-2.43	4.36E-02	Glutamate ionotropic receptor NMDA type subunit 1
INS	-2.40	8.32E-04	Insulin
SNRPG	-2.35	3.10E-02	Small nuclear ribonucleoprotein polypeptide G
PC	-2.34	1.19E-02	Pyruvate carboxylase
GTPBP3	-2.32	2.53E-02	GTP binding protein 3 (mitochondrial)
GTF2H4	-2.31	4.51E-02	General transcription factor IIH, polypeptide 4, 52kDa
NOTCH2	-2.21	2.50E-02	Notch 2
CHD5	-2.19	1.28E-02	Chromodomain helicase DNA binding protein 5
KIF2C	-2.17	1.41E-03	Kinesin family member 2C
CAV1	-2.05	5.24E-04	Caveolin 1, caveolae protein, 22kDa
PASK	-2.05	9.37E-03	PAS domain containing serine/threonine kinase
DKK1	-2.04	1.41E-02	Dickkopf WNT signaling pathway inhibitor 1
GHR	-2.03	1.74E-03	Growth hormone receptor
STMN2	-2.01	9.87E-04	Stathmin-like 2
IRF2	-1.95	1.17E-02	Interferon regulatory factor 2
